# Chimerization of human ESC-derived extraembryonic cells with the mouse blastocyst

**DOI:** 10.7150/ijbs.99519

**Published:** 2024-09-23

**Authors:** Siyi Fu, Borong Huang, Enqin Li, Xiaoling Xu, Ren-He Xu

**Affiliations:** Center of Reproduction, Development & Aging, and Institute of Translational Medicine, Faculty of Health Sciences, University of Macau, Taipa, Macau, China.

**Keywords:** Human embryonic stem cells, extraembryonic cells, trophoblasts, blastocyst injection, human-mouse chimera

## Abstract

It has been reported that human embryonic stem cells (hESCs) treated with BMP4 and inhibitors of TGFβ signaling (A83-01) and FGF signaling (PD173074), called BAP, can efficiently differentiate to extraembryonic (ExE) cells *in vitro*. Due to restricted access to human embryos, it is ethically impossible to test the developmental potential of ExE cells *in vivo*. Here, we demonstrate that most ExE cells expressed molecular markers for both trophoblasts (TBs) and amniotic cells (ACs). Following intra-uterine transplantation, ExE cells contributed to the mouse placenta. More interestingly, ExE cells could chimerize with the mouse blastocyst as, after injection into the blastocyst, they penetrated its trophectoderm. After implantation of the injected blastocysts into surrogate mice, human cells were found at E14 in placental labyrinth, junction zones, and even near the uterine decidua, expressed placental markers, and secreted human chorionic gonadotropin. Surprisingly, ExE cells also contributed to cartilages of the chimeric embryo with some expressing the chondrogenic marker SOX9, consistent with the mesodermal potential of TBs and ACs in the placenta. Deleting *MSX2*, a mesodermal determinant, restricted the contribution of ExE cells to the placenta. Thus, we conclude that hESC-derived ExE cells can chimerize with the mouse blastocyst and contribute to both the placenta and cartilages of the chimera consistent with their heteogenious nature. Intra-uterus and intra-blastocyst injections are novel and sensitive methods to study the developmental potential of ExE cells.

## Introduction

In 2002 we demonstrated that bone morphogenetic protein 4 (BMP4) triggers human embryonic stem cells (hESCs) to differentiate into trophoblasts (TBs) 1. This is a cell fate unexpected for hESCs as they are derived from the inner cell mass (ICM) of the human blastocyst 2 and the ICM is destined for the embryo proper but not TBs. The resultant TBs are featured by typical morphologies and markers of cytotrophoblasts (CTBs) and can further differentiate into syncytial TBs (STBs). Placental hormones including estradiol, progesterone, and human chorionic gonadotropin (hCG) are detected in the culture of the differentiated cells. Later, human induced pluripotent stem cells (hiPSCs) derived from somatic cells via reprogramming were also found to differentiate into TBs in response to BMP4 treatment 3. It is worth noting that, among BMP4-treated human pluripotent stem cells (hPSCs) including hESCs and hiPSCs, some marker genes such as *T*, *EOMES* and *TBX4* for the mesendoderm were also expressed 1, 3, 4. Deng and coworkers revealed that TBs together with cells representing the extra-embryonic endoderm are induced following BMP4 treatment for 7 days, whereas short-term (24 h) treatment of BMP4 results in mesodermal progenitor cells 5. They also uncovered that endogenous fibroblast growth factors (FGF) and transforming growth factor-beta (TGF-β)/Nodal/activin signaling are required for the mesodermal induction by BMP4. Consistently, Xiao and coworkers found that both inhibition of Activin/Nodal signaling and activation of the BMP signaling are required for hESC differentiation into TBs 6. Further, Thomson laboratory demonstrated that FGF2 maintains *NANOG* expression in hESCs through the MEK-ERK pathway, which switches the fate of BMP4-treated hESCs from TBs to the mesoderm 7.

Based on these findings, a combination of BMP4 and inhibitors of FGF and Activin signaling (PD173074 and A83-01), the so-called BAP formula, was developed and has been widely accepted to trigger the unidirectional differentiation of hESCs to TBs 3, 8-11. Over 97% TBs can be generated based on the ratio of cells positive for a TB marker cytokeratin 7 (KRT7), and the TBs can further differentiate into STBs and EVTs 3, 8-11. Such BAP-differentiated hESCs share great similarities with human primary TBs as follows. (1) KRT7, GATA3, and TFAP2C - three typical markers for human TBs - are all expressed by BAP-induced cells. (2) The gene profiling reveals that BAP-induced cells are closely related to CTBs isolated from human first-trimester placenta but distinguishable from the human placental stroma and amnion cells 12. (3) The methylation ratio of *ELF5* promoter is around 3.4% and 9.1% in CTBs and extra-villous TBs (EVTs), respectively, from the human first-trimester placenta; however, it reaches 87.5% in human placental mesenchymal cells. Consistently, the methylation ratio drops from 83.0% to 29.5% from hESCs to BAP cells 13, 14. (4) Like their primary counterpart, BAP-induced cells can differentiate into EVTs and STBs and the latter also possess invasive capability and hormone-secretion properties 8, 9. Interestingly, several groups have recently demonstrated that BAP-induced cells also express markers for extraembryonic amniotic cells (ACs) 15, 16. The amnion is a membrane containing two layers of cells from the extraembryonic ectoderm and extraembryonic mesoderm, respectively, and is a rich source for isolation of fetal mesenchymal stem cells (MSCs) 17. Theoretically, such MSCs are possibly derived from the amniotic mesoderm. Indeed, we have found that, under an MSC-inducing medium, hESCs treated with BMP4 and A83-01 (BA formula) can differentiate *in vitro* into MSCs (named T-MSCs with T standing for the intermediate TB-like stage) 18. Single-cell RNA sequencing (scRNA-seq) in this study demonstrates that BAP-induced cells mainly expressed TB markers while some expressing or co-expressing AC markers, thus we name the BAP-induced cells collectively as ExE cells in this study.

Regardless of the *in vitro* findings, a query remains unaddressed for whether ExE cells would really function *in vivo* as revealed *in vitro*. It has been widely demonstrated that chimera study is the gold standard for assessing and verifying the developmental potential of pluripotent cells. For example, the pluripotency of hPSCs was validated based on their extensive contributions into the mouse embryo following injection of hPSCs into the mouse blastocyst and such contributions were remarkably enhanced following the ectopic expression of an anti-apoptotic gene *BCL2* or *BMI1* 19, 20. We recently demonstrated that even multipotent cells such as T-MSCs can also chimerize with the mouse blastocyst, at least partially due to their high-level of BCL2, and mainly contribute to chondrogenic tissues in the chimera 21.

Although ExE cells are not pluripotent, we hypothesized that, since ExE cells originate in the blastocyst, they might also chimerize with the mouse blastocyst and contribute to the placenta. Indeed, we found here that ExE cells widely incorporated to the mouse placenta after intra-uterine transplantation. By injecting ExE cells into the mouse blastocyst we observed that the human cells embedded into the TE of the pre-implantation blastocyst and later contributed to the placenta following implantation into a surrogate mouse. Surprisingly, ExE cells were also found in some chondrogenic tissues derived from the embryonic mesoderm probably because ExE cells contained cells with mesodermal potential, which was prevented following knockout (KO) of the mesodermal determining gene *MSX2* (muscle segment homeobox 2) in ExE cells. Thus, we propose that ExE cells can chimerize with the mouse blastocyst and contribute to not only the placenta but also chondrogenic tissues in the embryo, indicating the heterogeneity of ExE cells.

## Results

### Transcriptomic characterization of ExE cells at the bulk and single-cell levels

**Figure 1:**
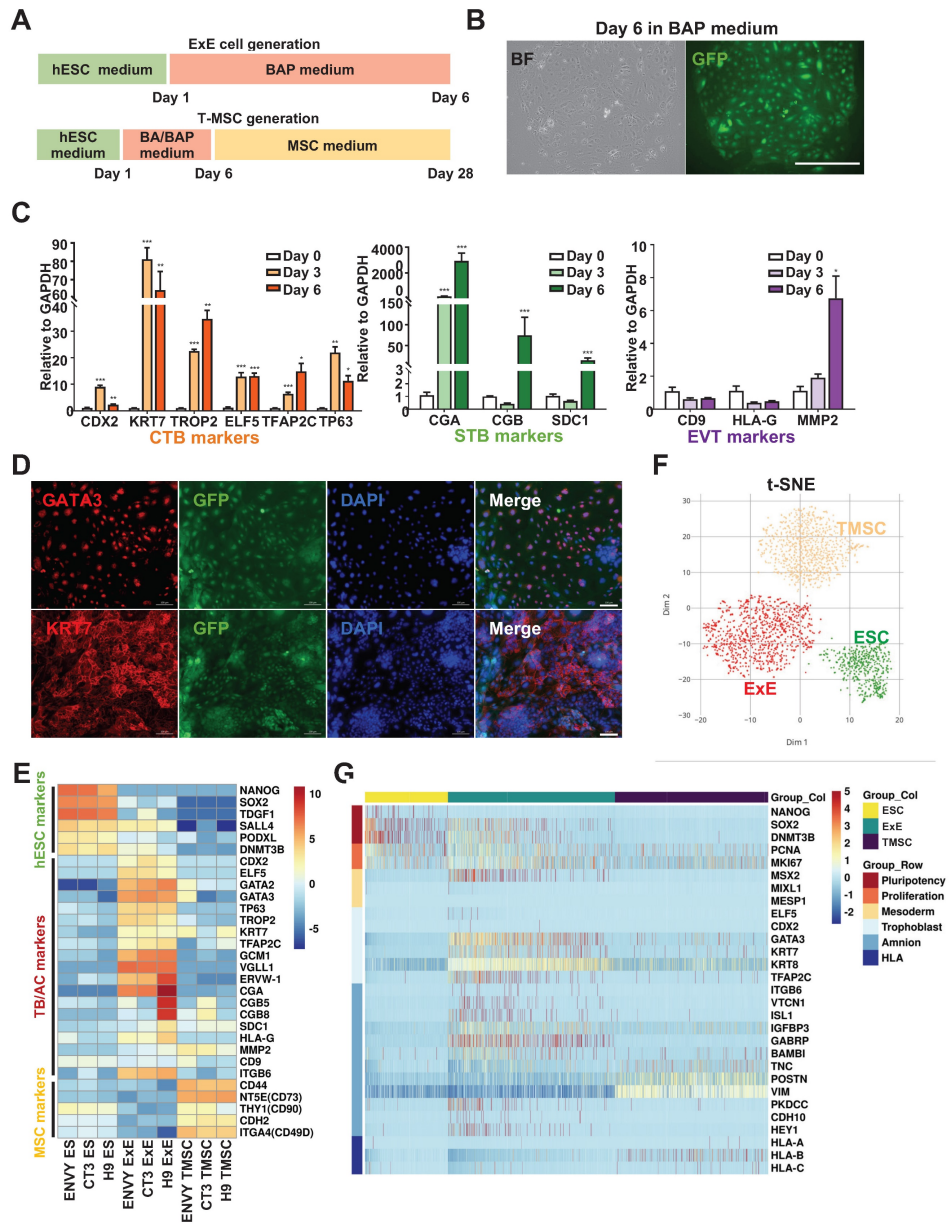
***In vitro* characterization of ExE cells. (A)** A schematic graph for hESC differentiation to ExE cells (designated TBs) and further differentiation to T-MSCs. **(B)** Bright-field (BF) and fluorescent (for GFP) images of ExE cells on day 6 of differentiation from the Envy hESC line. **(C)** QPCR detection of the expression of TB, STB, and EVT markers during hESC differentiation to ExE cells. *P < 0.05; **P < 0.01, and ***P < 0.001. N = 4. **(D)** Immunostaining for GFP and TB markers GATA3 and KRT7. DAPI counterstained the cellular nuclei. Scale bar, 100 μm. **(E)** Heatmap for expression of hESC, TB and MSC marker genes during hESC differentiation to ExE cells. The data were obtained from RNA-seq of three hESC lines. **(F)** T-SNE plot for single cell RNA-seq of hESCs, ExE cells and T-MSCs. **(G)** Heatmap for ESC, proliferation, mesoderm, TB, amnion and HLA class genes of hESCs, ExE cells and T-MSCs.

ExE cells were generated via differentiation from hESCs using the designated formula 8 (Figure 1A). The Envy hESC line was first used as it constitutively expressed green fluorescent protein (GFP) for convenient tracing of their derivatives *in vivo* 22. By day 6 of the BAP treatment, hESCs differentiated into flattened epithelium-like cells (Figure 1B & S1A) which were positive for the TB markers GATA3 and KRT7 (Figure 1D). QPCR assay further demonstrated that the differentiating cells expressed CTB markers *KRT7*, *TROP2*, *ELF5*, *TFAP2C*, and *TP63* in a time-dependent manner, suggesting the TB nature of ExE cells (Figure 1C). Some STB markers *CGA*, *CGB*, and *SDC1* and the EVT marker *MMP2* were also expressed in ExE cells (Figure 1C) and hCG was detected in the spent medium of ExE cells differentiated from the Envy as well as CT3 hESC lines (Figure S1B), indicating simultaneous differentiation of ExE cells. We performed bulk RNA sequencing on three hESC lines (Envy, CT3, and H9) during their differentiation in the BAP formula and confirmed a gradual decline of pluripotency markers and increase of TB markers (Figure 1E) consistent with the early reports 3, 8. All the hESC lines used in chimera study were each confirmed to possess a normal karyotype (Figure S1C & D).

To clarify whether ExE cells also include other cell lineages especially ACs as debated in the literature 15, 16, we differentiated hESCs into T-MSCs as described above 18, followed by scRNA-seq on hESCs, ExE cells, and T-MSCs (Figure 1A). The results demonstrate that ExE cells included a major cluster of TBs, clearly distinguished from the clusters of hESCs and T-MSCs (Figure 1F). ExE cells mainly expressed TB markers *GATA3*, *TFAP2C*, *KRT7*, and *KRT8*, while some expressed or coexpressed AC markers *ISL1*, *ITGB6*, and *VTCN1*, and *GABRP* (Figure S1E & F). It's worth noting that T-MSCs also expressed some of the AC genes at even much higher levels than ExE cells (Figure 1G). These data suggest that ExE cells may include not only TBs as the majority but also a minor group of cells with the AC potential.

**Figure 2:**
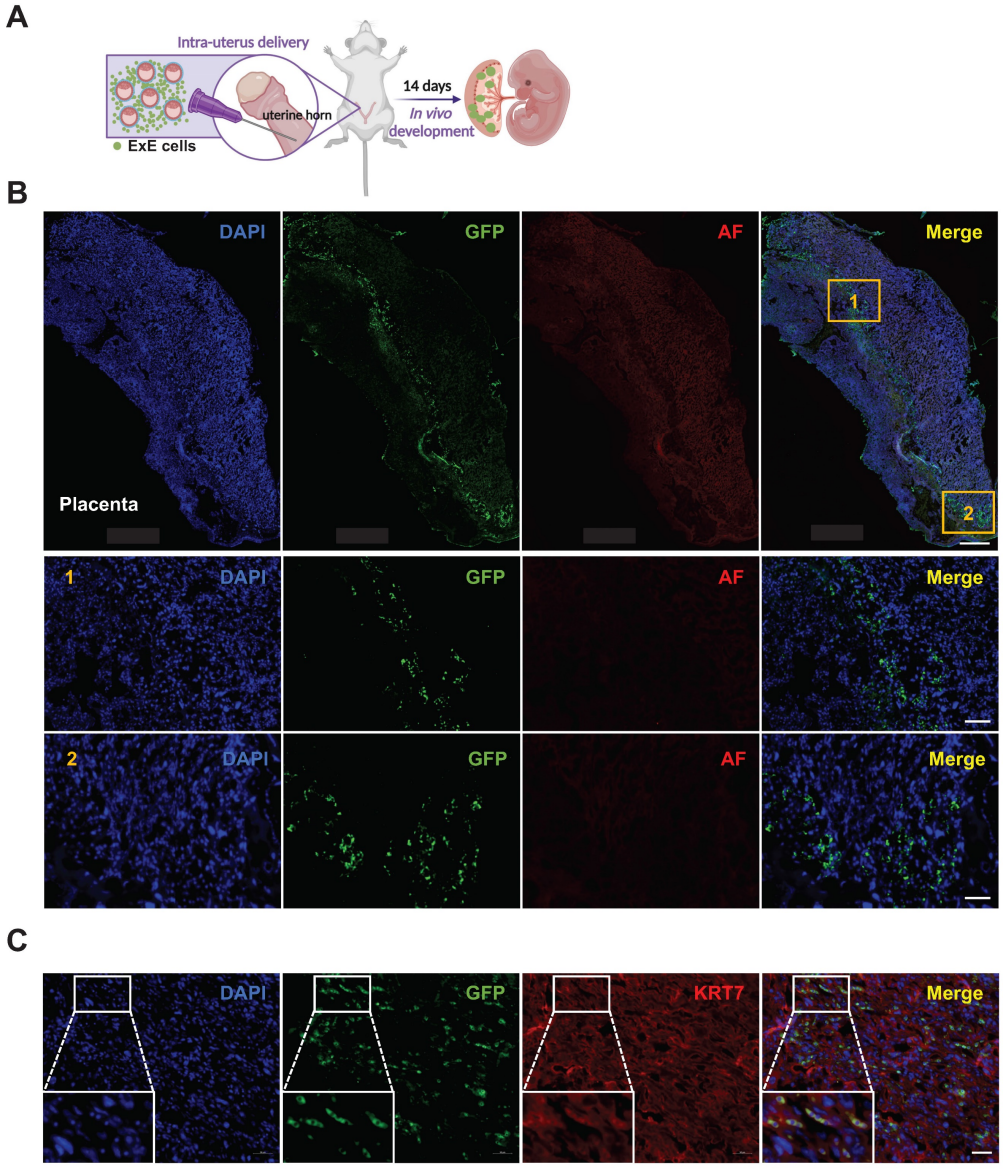
** Contribution of ExE cells to the mouse placenta but not embryo following intra-uterus delivery. (A)** A schematic graph for delivery of ExE cells together with murine blastocysts into the mouse uterus. **(B)** Immunostaining for GFP and auto fluorescence (AF) imaging as a control for ExE cells in a mouse placenta at E14. DAPI stained cellular nuclei. Two marked areas are enlarged below. Scale bar, 500 μm for the upper panel and 100 μm for the bottom panel. **(C)** Immunostaining for GFP to track ExE cells and for KRT7 to indicate TBs in a mouse E14 placenta. Scale bar, 50 μm.

To study the developmental potential of ExE cells, we delivered the cells together with freshly isolated mouse blastocysts into the uterus of surrogate mice. By embryonic day 14 (E14), embryos were harvested and sectioned for detection of GFP^+^ cells (Figure 2A, S2A & B). Numerous GFP^+^ cells were found in the mouse placenta (Figures 2B & C) but not the fetus (Figures S2C). This result demonstrates that ExE cells can contribute to the placenta following intra-uterine transplantation.

### ExE cells incorporate into the TE of the mouse blastocyst followed by *in vitro* development

**Figure 3:**
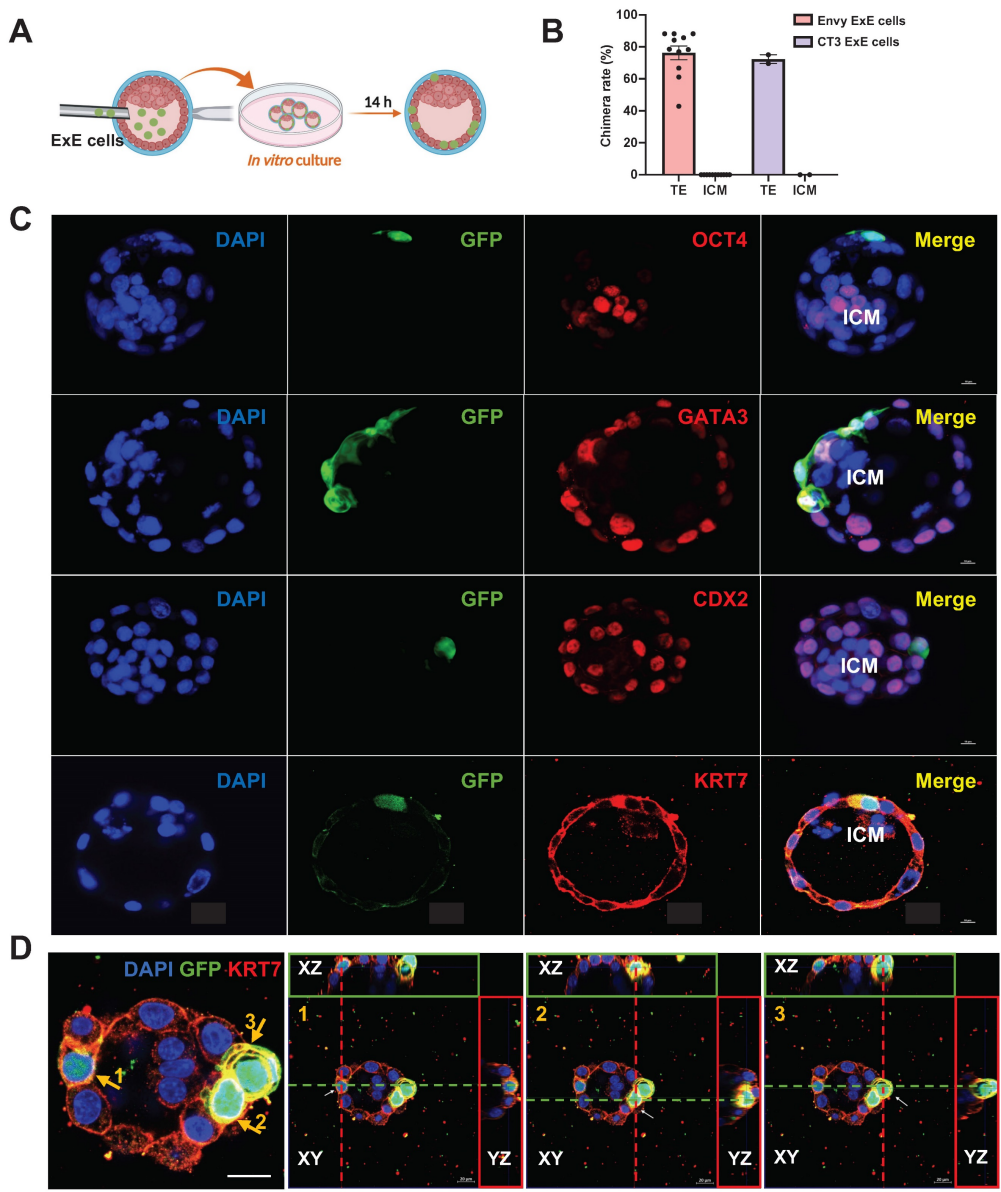
** Distribution and fate determination of ExE cells after injection into mouse blastocysts and extended culture *in vitro*. (A)** A schematic graph for injection of ExE cells into mouse blastocysts followed by *in vitro* culture. **(B)** A bar chart based on the data above to display the chimera rate, i.e., the ratio of mouse embryos with ExE cells contributed into the TE or ICM among all injected embryos. **(C)** Immunostaining of injected embryos for the ICM marker OCT4, TE markers GATA3, CDX2, and KRT7, and GFP for ExE cells. Scale bar, 50 μm. **(D)** Immunostaining of a injected embryos for the TE marker KRT7 and GFP for ExE cells with DAPI counterstaining the cellular nuclei. Three KRT^+^/GFP^+^ cells in the TE are highlighted in next three individual images, respectively, each visualized along the XY, XZ, and YZ axes. Scale bar, 20 μm.

Considering the potential of developmental compatibility of human ExE cells with the mouse blastocyst, we injected ExE cells derived from hESCs directly into the mouse blastocyst to test their fate choice between the TE and ICM. The injected embryos were transferred to IVC1 medium for further culture and the injected cells were traced for their status and migration in the blastocysts developing *in vitro* (Figure 3A). GFP^+^ cells were present randomly in the cavity right after the injection and then migrated into the TE after culture (Figure 3B & C). To determine the location of the injected ExE cells, we immunostained the embryos for GFP to label the human cells, OCT4 for the ICM, and CDX2, KRT7, and GATA3 for the TE. GFP^+^ cells were uniformly located in the TE but excluded from the ICM, and co-expressed CDX2, KRT7, and GATA3, indicating the presence of TBs among ExE cells (Figure 3C). Z-stack scanning for three-dimensional (3D) imaging demonstrates that three marked GFP^+^ cells clearly imbedded into the TE (Figure 3D). Similar phenomena and chimerization ratio (over 70%) were observed with ExE cells derived from both Envy and CT3 hESC lines (Figures 3B, S3A & B). These results indicate that the major destination of the injected ExE cells was the TE in the mouse blastocyst.

### ExE cells contribute to the placenta and embryo of E14 chimeras

**Figure 4:**
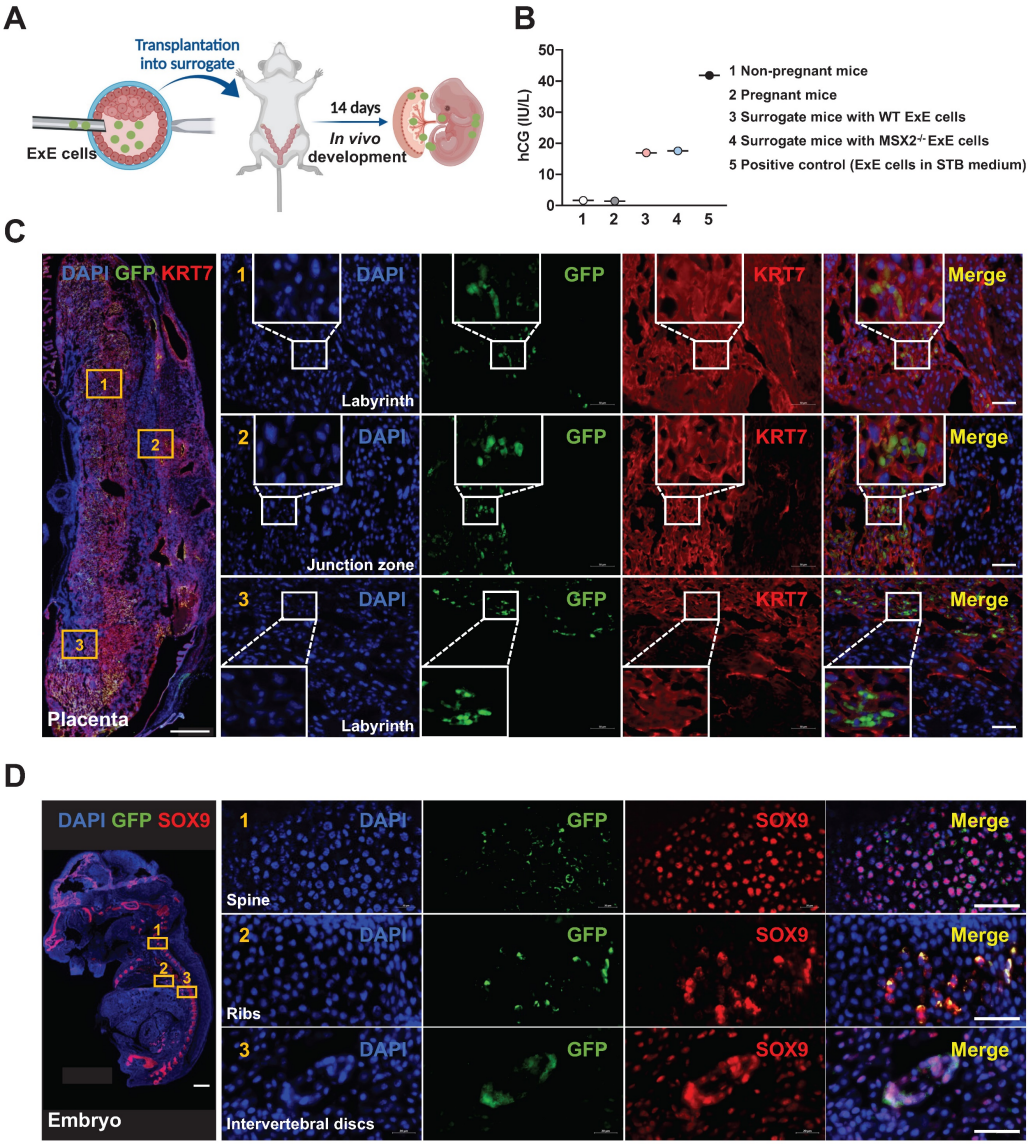
** Contribution of ExE cells to the mouse placenta and embryo at E14. (A)** A schematic graph for transplantation of ExE cells-injected mouse embryos into the uterus of surrogate mice. **(B)** Detection of hCG in the peripheral blood of transplanted and control pregnant mice. **(C)** Immunostaining for GFP (to track the derivatives of ExE cells) and KRT7 for TBs in a chimeric placenta at E14. Scale bar, 500 μm for the left panel and 50 μm for the right panels to enlarge three marked areas. **(D)** Immunostaining for GFP and SOX9 (for chondrogenic cells) in a chimeric embryo at E14. Scale bar, 500 μm for the left panel and 50 μm for the right panels to enlarge three marked areas.

To analyze the developmental potential of ExE cells in the chimeric embryos developing *in vivo*, we transplanted some injected blastocysts to the uterus of surrogate mice and allowed the embryos to continue the development. By E14 the embryos were collected to determine human cell contributions to various tissues (Figure 4A). GFP^+^ cells were found in some placental labyrinth areas near the junction zone and some even near the uterine decidua (Figure 4C). Besides, almost all the cells were also positive for the TB marker KRT7 (Figure 4C), indicating that the cells were destined along the TB fate. These cells were confirmed positive for the human nuclear antigen (HNA) (Figure S4A). Consistently, hCG was detected in the serum from the surrogate mice, demonstrating the existence and function of human STBs (Figure 4B).

Surprisingly, some tissues in the embryo including the spine and intervertebral discs (which are all derivatives from the embryonic mesoderm) also contained GFP^+^ cells, many of which co-expressed the chondrogenic factor SOX9 23, consistent with their location in the cartilages (Figure 4D & S4B). These results suggest that cells with mesodermal potential among the ExE cell population can contribute to the chondrogenic lineages.

To quantify the human cell contributions, we collected various tissues from the chimeras at E14 for DNA extraction. Human genomic DNA, represented by the amount of human thymidine kinase (*hTK*) gene per tissue, was detected in both the placenta and embryo of the samples, with the human/mouse cell ratio ranging from 0.0574% to 0.0669% (0.0599% in average) (Figure 6C) 24. The chimerization ratio calculated via detection of the *hTK* level is consistent with the results obtained through immunostaining.

### *MSX2* KO voids the mesodermal contribution while sustaining the placental contribution of ExE cells

**Figure 5:**
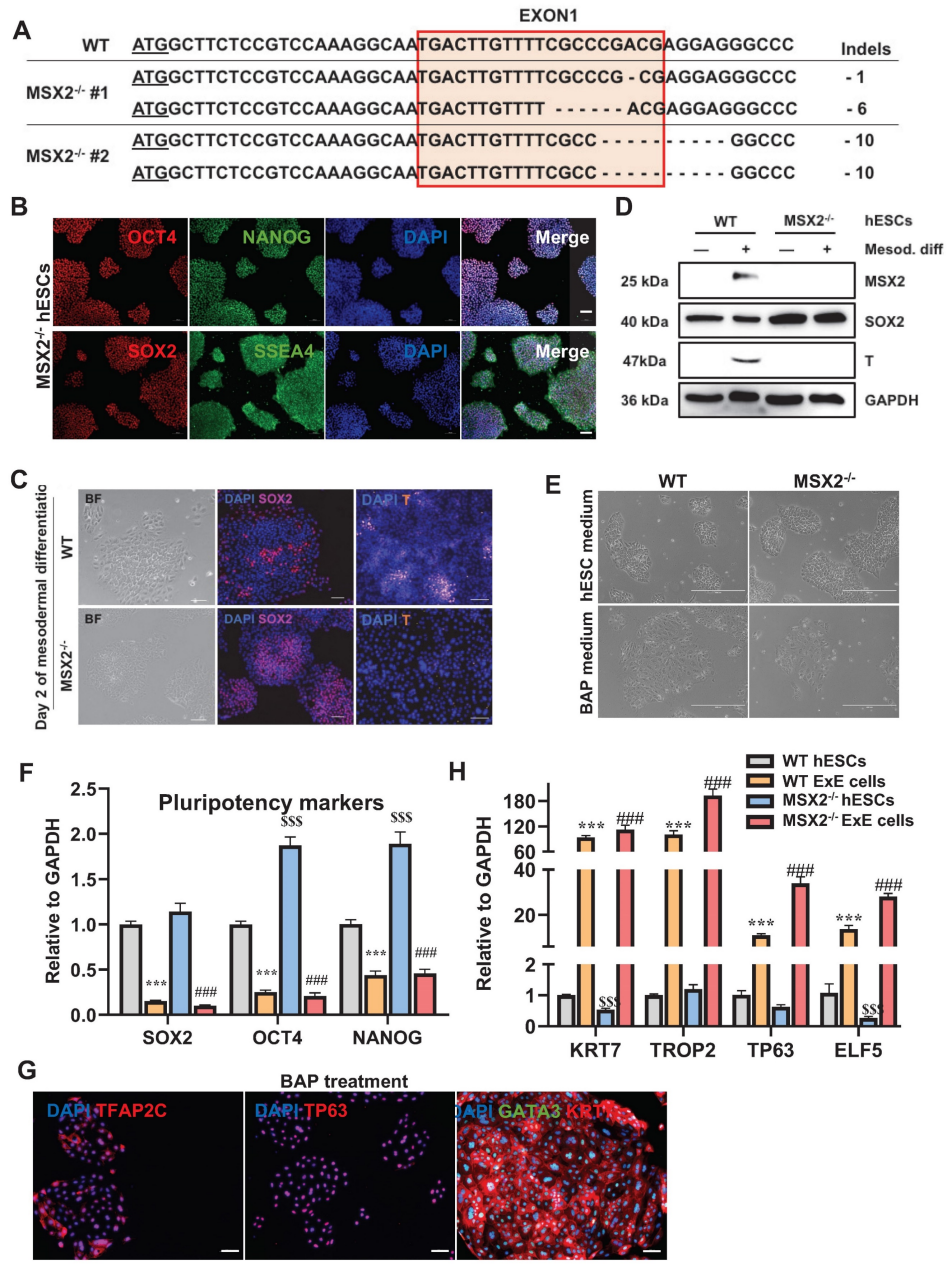
** Verification of the pluripotency of MSX2^-/-^ hESCs and enhanced ability to differentiate to TBs. (A)** Genotyping results of two MSX2^-/-^ hES cell clones. **(B)** Immunostaining for pluripotency makers SSEA4, NANOG, OCT4, and SOX2 in MSX2^-/-^ hESCs. Scale bar, 100 μm. **(C)** Bright-field imaging and immunostaining for pluripotency marker SOX2 and mesodermal marker T in WT and MSX2^-/-^ hESCs following mesodermal differentiation for 2 days. Scale bar, 100 μm. **(D)** Western blotting for MSX2, SOX2, and T in WT and MSX2^-/-^ hESCs following mesodermal differentiation. GAPDH was tested as a loading control. **(E)** Bright-field photography of WT and MSX2^-/-^ hESCs before and after BAP treatment for 6 days. Scale bar, 400 μm. **(F)** QPCR detection of pluripotency markers in WT and MSX2^-/-^ hESCs after BAP treatment for 6 days. *** and $$$, P < 0.001 compared with WT hESCs; ###, P < 0.001 compared with MSX2^-/-^ hESCs. N = 3. **(G)** Immunostaining for TB markers KRT7, TP63, GATA3, and TFAP2C on MSX2^-/-^ hESCs treated with BAP medium for 6 days. Scale bar, 100 μm. (H) QPCR detection of TB markers on WT and MSX2^-/-^ hESCs before and after BAP treatment for 6 days. *** and $$$, P < 0.001 compared with WT hESCs; ###, P < 0.001 compared with MSX2^-/-^ hESCs. N = 3.

To verify the mesenchymal cells in the chimeras were derived from mesodermal progenitors among ExE cells, we knocked out *MSX2*, a key mediator of the mesoderm differentiation 25, using the CRISPR-Cas9 system. Two *MSX2^-/-^* clones with different genotypes were obtained (Figure 5A), which remained positive for NANOG, OCT4, SOX2, and SSEA4 (Figure 5B & F), indicating *MSX2* KO didn't compromise the pluripotency. Upon induction of mesodermal differentiation, MSX2 was induced in the WT, but not the *MSX2^-/-^*, hESCs. It has been known that MSX2 suppresses the expression of the pluripotency gene *SOX2* through direct binding to the *SOX2* promoter, which forces hESCs to exit from pluripotency 25. Indeed, *SOX2* expression decreased in the WT but increased in the *MSX2^-/-^* cells under the mesodermal differentiation condition as tested (Figure 5D), which was confirmed via immunostaining (Figure 5C). The mesodermal marker gene *T* was also upregulated in the WT cells but absent in the *MSX2^-/-^* cells during the differentiation (Figures 5C & 5D). These data suggest that *MSX2^-/-^* hESCs failed to differentiate into mesoderm.

**Figure 6:**
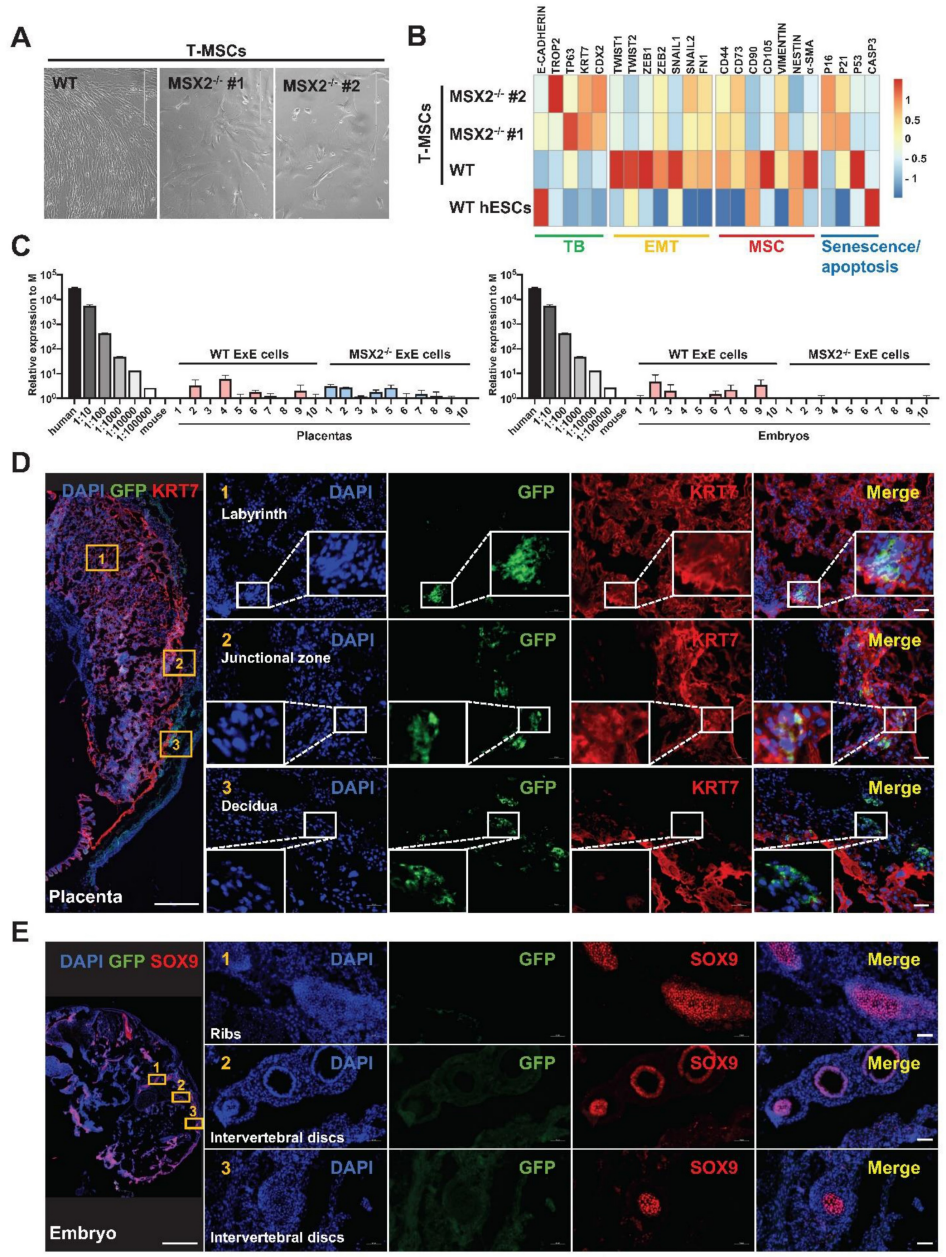
** MSX2 KO in ExE cells prevents their contribution to the embryo while sustaining their contribution to the placenta. (A)** Morphology of cells differentiated from wild-type (WT) and MSX2^-/-^ hESCs under the T-MSC differentiation protocol. **(B)** Heatmap for expression of TB, EMT, MSC, and senescence/apoptosis markers during differentiation of WT and MSX2^-/-^ hESCs to MSCs via TBs. **(C)** Quantitative PCR-based detection of human genomic DNA (represented by hTK) in E14 mouse embryos and placentas injected with WT or MSX2^-/-^ ExE cells. Human DNA isolated from hESCs and diluted at various ratios serve as a positive control, and mouse DNA isolated from a mouse embryo as a negative control. **(D)** Immunostaining for GFP (to track derivatives of ExE cells) and KRT7 (to label TBs) in a chimeric placenta at E14. Scale bar, 500 μm for the left panel and 50 μm for the right panels to enlarge three marked areas. **(E)** Immunostaining for GFP (to track derivatives of ExE cells) and SOX9 (to label chondrogenic cells) in a chimeric embryo at E14. Scale bar, 500 μm for the left panel and 50 μm for the right panels to enlarge three marked areas.

In contrast, *MSX2* KO didn't affect the TB-differentiation capability of hESCs. Under the BAP treatment, *MSX2^-/-^* hESCs could still differentiate into TBs with morphological changes like those observed with the WT control (Figure 5E). QPCR analysis confirmed the TB differentiation reflected by the downregulation of the pluripotency marker genes *OCT4*, *NANOG*, and *SOX2*, and up-regulation of the TB marker genes *KRT7*, *TROP2*, *TP63*, and *ELF5* in both WT and MSX2^-/-^ cells (Figure 5F & H). Immunostaining confirms that both differentiated WT and *MSX2^-/-^* cells were positive for GATA3, TFAP2C, and TP63 in the nucleus and KRT7 in the cell membranes (Figure 5G). Compared with the WT control, *MSX2^-/-^* cells even had higher transcription of *OCT4* and *NANOG* before differentiation (Figure 5F) consistent with the role of MSX2 in repressing pluripotency. More interestingly, under TB differentiation, *MSX2^-/-^* cells had higher expression of the TB marker genes than the WT control (Figure 5H). Interestingly, MSX2 can prevent CTBs from further differentiation to STBs 26, indicating its stage-dependent role. On the other hand, WT ExE cells could further differentiate into MSCs (as described above) but *MSX2^-/-^* ExE cells failed to do so (Figure 6A). QPCR analysis also indicates that *MSX2^-/-^* ExE cells were incapable of epithelial-mesenchymal transition (EMT) and mesenchymal differentiation based on the much lower expression of the marker genes for these processes than in the WT control (Figure 6B). Instead, *MSX2^-/-^* ExE cells had higher expression of the TB marker genes and the senescence genes *P16* and *P21* than the WT control (Figure 6B), indicating that the TBs with *MSX2* KO entered a senescent state. These results highly suggest that the mesodermal and TB fates compete during hESC differentiation and MSX2 is a key switch to induce the mesoderm in the expense of TBs.

Next, we injected *MSX2^-/-^* ExE cells into the mouse blastocyst. At E14, GFP^+^ cells were found in the mouse placenta including both labyrinth areas and the junction zone, overlapping with KRT7 indicating the fate of CTBs, and some GFP^+^ cells even invaded close to the maternal decidua (Figure 6D), indicating the fate of EVTs. In addition, hCG was detected in the serum of the surrogate mice for the transplanted blastocysts injected with either WT or *MSX2^-/-^* ExE cells, indicating the functionality of STBs (Figure 4B). In contrast, no GFP^+^ cells were found in tissues containing Sox9^+^ cells and anywhere else in the embryos injected with *MSX2^-/-^* ExE cells (Figure 6E). These results further suggest that ExE cells possess dual fate depending on their location and *MSX2* KO deprived the mesodermal fate while retaining the TB fate. Consistently, *hTK* DNA was detected only in the placenta but not the embryo of E14 chimeras formed by *MSX2^-/-^* ExE cells (Figure 6C).

## Discussion

In this paper, we recharacterized ExE cells via scRNA-seq and qPCR, which demonstrate that ExE cells not only include TBs as the majority but also a minor group of cells with the amniotic mesodermal potential. Then, by employing the chimeric systems, we characterized the fate of ExE cells *in vivo*. Intra-uterine transplantation of ExE cells together with mouse blastocysts into surrogate mice allowed the chimerization of ExE cells with the mouse placenta. Injection of ExE cells into the mouse blastocyst allowed their chimerization. ExE cells contributed to both the placenta and chondrogenic tissues in the embryo. Since the hESC lines used in this study were verified to each carry a normal karyotype (Figure S1 C& D), there is high possibility that our findings resulted from specific chimerism instead of mosaicism of genetically mutated cells trashed in the placenta 27.

It has been a long mystery how hESCs (derived from the ICM), which are in the primed state of pluripotency 28, can differentiate into ExE cells and whether these ExE cells possess corresponding properties. Although the ExE cells mainly manifest markers and functions of TBs including STBs *in vitro*, controversial results have been observed that BMP4-treated hESCs also express mesodermal markers 1, especially in the context of elevated FGF signaling 7. We have previously demonstrated that hESCs that have differentiated in the presence of BMP4 and A83-01 and absence of FGF2, somehow similar to the formula for ExE cells, can further differentiate to MSCs (T-MSCs) in a MSC medium 18, 21. In this study we demonstrate that ExE cells, although carrying typical TB hallmarks *in vitro*, also have a fate choice in the embryonic environment, depending on the context they reside. At early time, ExE cells penetrated the TE but not the ICM as shown in the injected blastocysts, resulting in ~70% chimera formation, which strongly suggests the cell preference in the environment. Such phenomena have also been discovered in chimera formed through injection of mouse TB stem cells into the mouse blastocyst 29, 30. However, after implantation into the uterus, ExE cell-injected blastocysts developed to embryos that, by E14, contained GFP^+^ cells in not only the placenta but also chondrogenic tissues in the embryo.

For ExE cells that contributed to the placenta, they mainly expressed the TB marker KRT7, secret hCG into the serum of surrogate mice, and invaded near the uterine decidua, indicating that ExE cells contained TBs which probably even differentiated into STBs and EVTs. However, due to the great differences in the structures and physiological functions between murine and human placentas, it is hard to analyse how a chimeric placenta is formed via the mutual influence and adaptation between the invading human TBs and host extraembryonic tissues. For example, the functional villus unit, which is responsible for nutrient uptake and gas exchange, has distinguished structures between human and mouse placenta. It is made up of continuous arrangement of CTBs together with one outer layer of STBs in humans, while in the mouse it has two layers of STBs and the cells inside are a discontinuous layer called sinusoidal TB giant cells (TGCs). In addition, human placental villus has fetal blood vessels inside whereas, in the mouse, foetal blood vessels are adjacent to the two layers of the villus 31-33. Thus, it remains to define whether human TBs participate in the foetal-maternal interface and how they function in the chimeric placenta. A compensation assay on defected mouse placenta may help address these questions.

It is puzzling how ExE cells contributed to the embryo proper, which was blocked following *MSX2* KO. One possibility is that some TBs among injected ExE cells may undergo epithelial-mesenchymal transition to become mesenchymal cells as Yamakoshi, *et al.*, showed *in vivo* 34 and we showed *in vitro* 35. MSC generation from TBs can be blocked by *MSX2* knockdown 36. Another possibility is that ACs among ExE cells possess mesenchymal potential 37. A recent study confirmed the capability of both hPSC-derived TBs and ACs to differentiate into MSCs 38. It remains to understand why ExE cells mainly differentiated to chondrogenic (SOX9^+^) cells in cartilage tissues of the chimeric embryos but not other mesodermal/mesenchymal tissues such as adipocytes, smooth muscles, and vascular endothelial cells - the lineages MSCs can easily differentiate into *in vitro*. Our results may implicate a real or major developmental path and destination of mesenchymal cells in the specific embryonic environment. Or it may take time for ExE cells to further contribute to more mature tissues at later developmental stages.

Together, this study demonstrates that (1) ExE cells include not only a major group of TBs but also a minor group of ACs and both have mesodermal potential; (2) the intra-uterus and intra-blastocyst injections are sensitive methods to study the developmental potential of ExE cells.

### Limitations

(1) Due to the limited cell numbers of ExE cells observed in the chimeric placenta and embryo, we were not able to harvest the human cells back for further analysis such as scRNA-seq.

(2) A suitable mouse placental defect model is needed to test whether human ExE cells can compensate for such defects.

(3) Given the great differences in the structures and functions between mouse and human placenta, it was difficult to test and distinguish the exact functions of human ExE cells in the chimera.

## Materials and Methods

### Ethics statement

This work was conducted under the University of Macau Research Ethics Panel protocol #BSERE19-APP026-FHS (for human ethics) and UMARE-030-2019 (for animal use ethics). It was also in accordance with the Guidelines for Stem Cell Research and Clinical Translation of the International Society for Stem Cell Research.

### Mice

C57BL/6, FVB, and ICR mouse strains used in this study were obtained from the animal facility of the University of Macau. All mice were maintained in the animal research core and free of specific pathogens per testing.

### Blastocyst preparation, microinjection, and embryo implantation

3-week-old female C56 or FVB were injected intraperitoneally (i.p.) with 5 IU pregnant mare serum gonadotropin (PMSG, Sigma, G4877) per mouse at 5~6 pm for superovulation. Human chorionic gonadotropin (hCG, Sigma, C1063) was injected i.p. into the same female mice 46~48 h later and the mice were mated to male mice of the same strain for overnight. Next day morning, plugs were checked before 10am and plug-positive female mice (E0.5) were sacrificed three days later (E3.5). ICR surrogate female mice were mated to vasectomized male mice one day later than these super-ovulated mice and plugs were also checked next morning. The male mice were from a mixed strain through crossbreeding among FVB, C57, and ICR to improve the mating capability.

M2 medium (Sigma, M7167) was used to flush out embryos from the uterus of the sacrificed female mice. Only blastocyst-stage embryos with big cavity were picked for microinjection. The cells for microinjection were dissociated into single cells and put on ice during the whole procedure.10-15 cells were injected into each blastocyst and injected embryos were cultured *in vitro* for overnight in the IVC1 medium containing 20% FBS, glutaMax (Invitrogen, 35050061), penicillin, streptomycin, ITS-X supplement, β-estradiol (Sigma, E8875), progesterone (Sigma, P0130), and N-acetyl-L-cysteine in DMEM/F12. Then, injected embryos were transplanted into the uterus of surrogate mice and allowed to develop to E14.

### Intra-uterine transplantation

During the transplantation stage, 1 x 10^4^ ExEcells were delivered together with wildtype fresh-separated mouse blastocysts (10 embryos each side) into the uterus of surrogate mice. Mice were allowed to develop to E14, and embryos and placentas were collected for further analysis.

### Culture of hESCs

Three hESC lines including Envy (GFP^+^)(P88), H9(P55), and CT3(P50), were used in this study. The cells were cultured on Matrigel-coated plate in mTeSR™1 medium (STEMCELL Technologies, 5870) under 20% O_2_ and 5% CO_2_ at 37 °C. Spent medium was refreshed every day and cells were passaged with EDTA/DPBS (Sigma, E5134) every 4-5 days 39. All the cells used in this study were Mycoplasma free and were tested once a month.

### Differentiation of hESCs to ExE cells

hESCs were dissociated into single cells with EDTA/DPBS and seeded in a 6-well plate with a density of 1.2 × 10^4^ cells/ml on day 0. Next day, mTeSR™1 medium was changed to the BAP medium containing 20% KnockOut™ Serum Replacement (KOSR) (Gibco, 10828028), Recombinant Human BMP-4 Protein (10 ng/mL, R&D systems, 314-BP-500/CF), A83-01 (1 μM, Sigma, SML0788), and PD173074 (0.1 μM, SELLECK, S1264) in DMEM/F12 medium (Gibco, 11330057). The BAP medium was refreshed every two days and cells were collected at designated times for analyses.

### Differentiation of hESCs to mesodermal cells

hESCs were split into colonies on day 0 with mTeSR1. Next day, cells were washed with DPBS twice and cultured in the BFW medium containing 10 ng/mL BMP4, 10 ng/mL bFGF and 1μM CHIR99021. The medium was refreshed every day and cells were collected at designated times for analyses.

### Differentiation of hESCs to T-MSCs

hESCs were firstly differentiated to ExE cells in the BA medium for 5 days (or BAP medium for 3 days) and then the medium was changed to a MSC medium containing 20% FBS in αMEM (Thermo, 12571063) for 20 days or so to differentiate to MSCs, named T-MSCs 18. The cells were characterized for MSC markers using the Human MSC characterization kit (BD, 562245) and confirmed for the capability of tri-lineage differentiation 40 according to the minimal criteria for defining MSCs of the International Society for Cell and Gene Therapy 41.

### Lentiviral packaging and transduction

For lentivirus packaging, 293T cells were split into a 6-well plate at around 50~70% confluence on day 1. Next day, Lipofectamine 3000 transfection reagent (Thermo, L30000015) was used for transfection of three plasmids including the lentiviral packaging plasmid pCMVR8.74 (Addgene, 22036), lentiviral envelope plasmid pMD2.G (Addgene, 12259), and the target plasmid pSin-EF2-GFP-Puro (modified from Addgene, 16578) at a ratio of 1:1:1. The medium in the transfected cells was refreshed after 8-12 h, and the supernatant was collected at 48 and 72 h. The supernatant containing GFP lentivirus was combined and filtered through a sterilized 0.45-micron filter. Target cells were then transduced with the GFP lentivirus together with polybrene (10 μg/ml, Sigma, TR1003) and the spent medium was refreshed after 24 h. The transduced cells were selected for stable clones with puromycin (100 ng/ml, ACROS, 58-58-2) for at least one week.

### Genome editing

MSX2 sgRNA oligos and primers were all synthesized by BGI Genomics. Two pairs of sgRNA were annealed, respectively, to generate sgRNA1 and sgRNA2. Each annealed sgRNA was inserted into the BbsI site of the pX458-Ef1a-Cas9-H2B-GFP plasmid (Addgene, 159654) which carried genes encoding Cas9 and GFP (for selection). The constructed plasmids were transfected into 293T cells and T7EI assay was performed to validate the efficiency of sgRNAs.

hESCs were transfected with sgRNA plasmid using Lipo3000 and the transfected cells were sorted for GFP^+^ cells using FACSAria III equipment (BD Biosciences). Single clones expanded from the sorted cells were monitored under Incucyte (Sartorius) and picked for expansion. Genomic DNA was extracted from each picked clone for genotyping with the DNA extraction kit (Tiangen, DP304). Fragments containing the sgRNA targeting site were amplified via PCR using genotyping forward/reverse primers and the Q5 polymerase (NEB, M0491). The PCR products were cloned into T-vectors using pEASY®-T1 Cloning Kit (TransGen Biotech, CT101-01) and multiple single colonies were picked for Sanger-sequencing at BGI Genomics.

### Histological analysis and immunostaining

Cells were fixed with 4% paraformaldehyde (PFA, Sigma, 158127) for 30 min., permeabilized with 0.1% Triton X-100 for 15 min. and blocked with 3% BSA for one h at room temperature. Then the cells were incubated with the primary antibodies at 1:200~500 dilution in cold room (4°C) overnight. Secondary antibodies were used at 1:500 dilution for incubation at room temperature for 2 h. Nuclei were counterstained with DAPI for 5 min. and images were taken under a fluorescence microscope (LSM710, Zeiss).

Mouse embryos were collected and fixed with 4% PFA overnight. 3% sucrose (FLUKA, 84100) was used for dehydration and then the embryos were embedded in O.C.T. Compound (Fisher Scientific) and then quickly placed at -80°C. The frozen embryos were cryo-sectioned at 10 µm and stained for designated antibodies and the nuclei were counterstained with DAPI. Images were taken under a fluorescence microscope (LSM710, Zeiss).

### Western blotting

Cells cultured in 6-well plates were collected and lysed using the radioimmunoprecipitation assay (RIPA) buffer (Invitrogen, 89900) containing the protease inhibitor cocktail (Invitrogen, 87786) at 1:1000 dilution and incubated on ice for 30 min. The supernatants (as cell lysates) were collected after centrifugation for 15 min. at 12,000 rpm and the protein concentration was quantified via the Bradford assay (BioRad, 5000201). The lysates were diluted into a target concentration for all the samples, which were then mixed with 4x Laemmli Sample Buffer (BioRad, 1610737). An aliquot of each sample containing 20 μg protein was loaded into each lane of a sodium dodecyl sulfate polyacrylamide gel electrophoresis gel (SDS-PAGE gel, BioRad, 3458166) for separation and separated protein bands on the gel were transferred to a polyvinylidene fluoride (PVDF) membrane (Millipore, IPVH00010) under the semi-dry transfer system (BioRad). The membrane was incubated with 3-5% Blotting Grade Blocker Non-Fat Dry Milk (BioRad, 170-6404) containing 1% PBST (for 1 h) followed by incubation with a primary antibody at cold room (4 ℃) for 12-16 h. After washing with PBST for three times, the membrane was incubated with a secondary antibody for 1 h at room temperature. Signals were visualized via enhanced chemiluminescence (ECL) (Bio-rad, 1705061) and images captured using ChemiDoc Imager (Bio-Rad).

### Real-time quantitative polymerase chain reaction (qPCR)

Total RNA was extracted from cells using Trizol (Thermo, 15596026). First-strand cDNA was synthesized from total RNA using the cDNA Reverse Transcription Kit (Thermo Fisher, 4368814) and qPCR was performed using iTaqTM Universal SYBR Green Supermix (Bio-rad, 1725124). The mRNA level for target genes was determined using the ΔΔCt method and normalized by the level of GAPDH as a loading control.

### Measurement of the hCG hormone

hESCs were induced to differentiate in the BAP medium with the spent medium refreshed every 2-3 days. The supernatants were collected at day 6 of the differentiation and stored at -80 °C. Spent medium collected from the culture of undifferentiated hESCs was used as negative control. The hCG level in the medium was measured using the HCG ELISA kit (Abnova, KA4005).

### RNA sequencing and analysis

Cells were collected and lysed in Trizol, and the total RNA was extracted as mentioned above and quickly frozen at -80°C until shipping out for sequencing. Data was analyzed on the Galaxy Project platform (https://usegalaxy.org). All data were aligned to the human genome (hg38.refGene) using HISAT2 (Galaxy Version 2.2.1). htseq-count (Galaxy Version 0.9.1) was applied for gene counts.

### Quantification and statistical analysis

All data was graphically illustrated using GraphPad Prism 8 (GraphPad, USA) and R software. They are presented as mean ± S.E.M. Statistical analyses were performed via t test using the SPSS (IBM SPSS 20). Statistically significant results were considered when *P* < 0.05 with the signs * for *P* < 0.05, ** *P* < 0.01, and *** *P* < 0.001.

### Data and code availability

The RNA sequencing data in this study are available in the Gene Expression Omnibus (GEO) database with the accession numbers of GSE224029 and GSE195573.

## Supplementary Material

Supplementary figures and tables.

## Abbreviations

hESC: human embryonic stem cell
TGFβ: transforming growth factor beta
FGF: fibroblast growth factors
ExE: extraembryonic
TBs: trophoblasts
ACs: amniotic cells
SOX9: sry-box transcription factor 9
MSX2: muscle segment homeobox 2
BMP4: bone morphogenetic protein 4
ICM: inner cell mass
TE: trophoectoderm
CTBs: cytotrophoblasts
STBs: syncytiotrophoblasts
EVTs: extravillous trophoblasts
hCG: human chorionic gonadotropin
hiPSC: human induced pluripotent stem cell
hPSC: human pluripotent stem cell
WT: wild-type
KO: knockout
MSCs: mesenchymal stem cells
T-MSCs: hESC-derived MSC through intermediate TB-like stage
CRISPR: clustered regularly interspaced short palindromic repeats
Cas9: CRISPR-associated protein 9
qPCR: quantitative PCR
GFP: egreen fluorescent protein
IVC1: *in vitro* culture 1
hTK: human thymidine kinase
