# Supplementary material for: Chimerization of human ESC-derived extraembryonic cells with the mouse blastocyst
Source: Int J Biol Sci. 2024 Sep 23;20(13):5056–69. doi: 10.7150/ijbs.99519 (PMC11488584; doi:10.7150/ijbs.99519)
Supplement: Supplementary file 1 — Supplementary figures and tables. [file ijbsv20p5056s1.pdf]

Supplemental Tables

Table S1. List of oligos and primers used in this study.

Name	Sequences
CDX2 forward primer	5’-GACGTGAGCATGTACCCTAGC-3’
CDX2 reverse primer	5’-GCGTAGCCATTCCAGTCCT-3’
KRT7 forward primer	5’- CATCGAGATCGCCACCTACC -3’
KRT7 reverse primer	5’- TGGAGAAGCTCAGGGCATTG -3’
ELF5 forward primer	5’- TAGGGAACAAGGAATTTTTCGGG-3’
ELF5 reverse primer	5’- GTACACTAACCTTCGGTCAACC -3’
TROP2 forward primer	5’- GGAAGGGATGGCATAGCGTT -3’
TROP2 reverse primer	5’- ACTGAGTTTCCGGGCAATGT -3’)
TFAP2C forward primer	5’- CTGTTGCTGCACGATCAGACA -3’
TFAP2C reverse primer	5’- CTGTTGCTGCACGATCAGACA -3’
TP63 forward primer	5’- GGACCAGCAGATTCAGAACGG -3’
TP63 reverse primer	5’- AGGACACGTCGAAACTGTGC -3’
HCGA forward primer	5’- TAAAGGGGATACCGAGGTGATG-3’
HCGA reverse primer	5’- TCGGAGTGTCTCAAAACATTCC -3’
HCGB forward primer	5’- ACCCTGGCTGTGGAGAAGG -3’
HCGB reverse primer	5’- ATGGACTCGAAGCGCACA-3’
SDC1 forward primer	5’- GCTGACCTTCACACTCCCCA -3’
SDC1 reverse primer	5’- CAAAGGTGAAGTCCTGCTCCC -3’
HLA-G forward primer	5’- GCCGGAGTATTGGGAAGAGG -3’
HLA-G reverse primer	5’- CCACTGGAGGGTGTGAGAAC -3’
MMP2 forward primer	5’-TGGCACCCATTTACACCTACAC -3’
MMP2 reverse primer	5’- ATGTCAGGAGAGGCCCCATAGA-3’
CD9 forward primer	5’- ATGTCAGGAGAGGCCCCATAGA -3’
CD9 reverse primer	5’- ATGTCAGGAGAGGCCCCATAGA-3’
MSX2 forward primer	5’- TTACCACATCCCAGCTCCTC-3’
MSX2 reverse primer	5’- CCTGGGTCTCTGTGAGGTTC-3’
SOX2 forward primer	5’-GCTACAGCATGATGCAGGACCA -3’
SOX2 reverse primer	5’- TCTGCGAGCTGGTCATGGAGTT -3’
OCT4forward primer	5’-GTGTTCAGCCAAAAGACCATCT-3’
OCT4 reverse primer	5’-GGCCTGCATGAGGGTTTCT-3’
NANOG forward primer	5’-TTTGTGGGCCTGAAGAAAACT-3’
NANOG reverse primer	5’- AGGGCTGTCCTGAATAAGCAG-3’
GAPDH forward primer	5’- AGGGCTGCTTTTAACTCTGGT -3’
GAPDH reverse primer	5’- CCCCACTTGATTTTGGAGGGA -3’
MSX2-sg1-F	5’-CACCGTGACTTGTTTTCGCCCGACGAGG-3’
MSX2-sg1-R	5’- AAACCCTCGTCGGGCGAAAACAAGTCAC -3’
Genotying1-F	5’-CACCGTGACTTGTTTTCGCCCGACGAGG-3’
Genotying1-R	5’- AAACCCTCGTCGGGCGAAAACAAGTCAC-3’

Table S2. List of antibody used in this study.

Antibody	Source	Identifier
FITC goat polyclonal anti-GFP	Abcam	ab6662; RRID: AB_305635
Rabbit polyclonal anti-GFP	Invitrogen	A-6455; RRID: AB_221570
Rabbit polyclonal anti-Cytokeratin 7	Abcam	ab181598; RRID: AB_2783822
Rabbit Polyclonal anti-MSX2	Invitrogen	PA5-40367; RRID: AB_2608897
Rabbit polyclonal anti-SOX2	Abcam	Ab97959; RRID: AB_2341193
Goat Polyclonal anti-Brachyury(T)	Invitrogen	PA5-46984; RRID: AB_2610378
Rabbit polyclonal anti-OCT4	Invitrogen	PA5-27438; RRID: AB_2544914
Mouse monoclonal anti-Gata3	Thermo Fisher	MA1-028; RRID: AB_2536713
Rabbit polyclonal anti-Sox9	Millipore	AB5535; RRID: AB_2239761
Rabbit polyclonal anti-TP63	BOSTER	BA1887; RRID: NA
Rabbit polyclonal anti-TFAP2C	Sigma	AV38284; RRID: AB_1857918
Goat anti-Rabbit IgG (H+L) Cross-Adsorbed Secondary Antibody, HRP	Invitrogen	G-21234; RRID: AB_2536530
Goat anti-Mouse IgG (H+L) Cross-Adsorbed Secondary Antibody, HRP	Invitrogen	G-21040; RRID: AB_2536527
Mouse monoclonal anti-GAPDH	Abclonal	AC002; RRID: AB_2736879
Rabbit monoclonal anti-β-actin	Abclonal	AC038; RRID: AB_2863784
Donkey anti-Mouse IgG (H+L) Highly Cross-Adsorbed Secondary Antibody, Alexa Fluor 488	Invitrogen	A-21202; RRID: AB_141607
Donkey anti-Rabbit IgG (H+L) Highly Cross-Adsorbed Secondary Antibody, Alexa Fluor 488	Invitrogen	A-21206; RRID: AB_2535792
Goat anti-Rabbit IgG (H+L) Cross-Adsorbed Secondary Antibody, Alexa Fluor 546	Invitrogen	A-11010; RRID: AB_2534077
Donkey anti-Mouse IgG (H+L) Highly Cross-Adsorbed Secondary Antibody, Alexa Fluor 594	Invitrogen	A-21203; RRID: AB_141633

Donkey anti-Rabbit IgG (H+L) Highly Cross-Adsorbed Secondary Antibody, Alexa Fluor 594	Invitrogen	A-21207; RRID: AB_141637
Donkey anti-Rabbit IgG (H+L) Highly Cross-Adsorbed Secondary Antibody, Alexa Fluor 647	Invitrogen	A-31573; RRID: AB_2536183
Donkey anti-Mouse IgG (H+L) Highly Cross-Adsorbed Secondary Antibody, Alexa Fluor 647	Invitrogen	A-31571; RRID: AB_162542

Table S3. List of chemicals, peptides, and recombinant proteins used in this study.

**Chemicals, peptides, and recombinant proteins**	**Source**	**Identifier**
KnockOut™ Serum Replacement	Thermo	10828028
BMP4	R&D	314-BP
PD173074	Stem cell	72164
A8301	Stemgent	04-0014-2
PMSG	Sigma	G4877
hCG	Sigma	C1063
M2 medium	Sigma	M7167
α-MEM	Gibco	12571071
DMEM/F12	Gibco	11330057
Progesterone	Sigma	P0130
β-estradiol	Sigma	E8875
GlutaMax	Invitrogen	35050061
NEAA	Gibco	11140050
CHIR 99021	SelleckChem	S2924
2-Mercaptoethanol	Sigma	63689
Puromycin	ACROS	58-58-2
Rock inhibitor, Y27632	Tocris	1254
Penicillin-Streptomycin	Thermo	10378016
TrypLE	Thermo	12605010
mTeSR1 medium	Stem Cell Technologies	5870
EDTA	sigma	E5134
DPBS	Gibco	14190250
4% Paraformaldehyde (PFA)	Sigma	158127
Sucrose	FLUKA	84100
FBS	Gibco	26140079
BSA	Sigma	A7030
Triton X-100	Sigma	93443
Tyrode's solution	Sigma	T1788
4’, 6-diamidino-2-phenylindole (DAPI)	Sigma	D9542
Critical commercial assays		

RIPA buffer	Thermo Scientific	89901
Protease inhibitor cocktail	Sigma	P-8465
Bovine serum albumin standard set	Bio-rad	500-0207
Western ECL Substrate	Bio-rad	170-5061

Figure S1

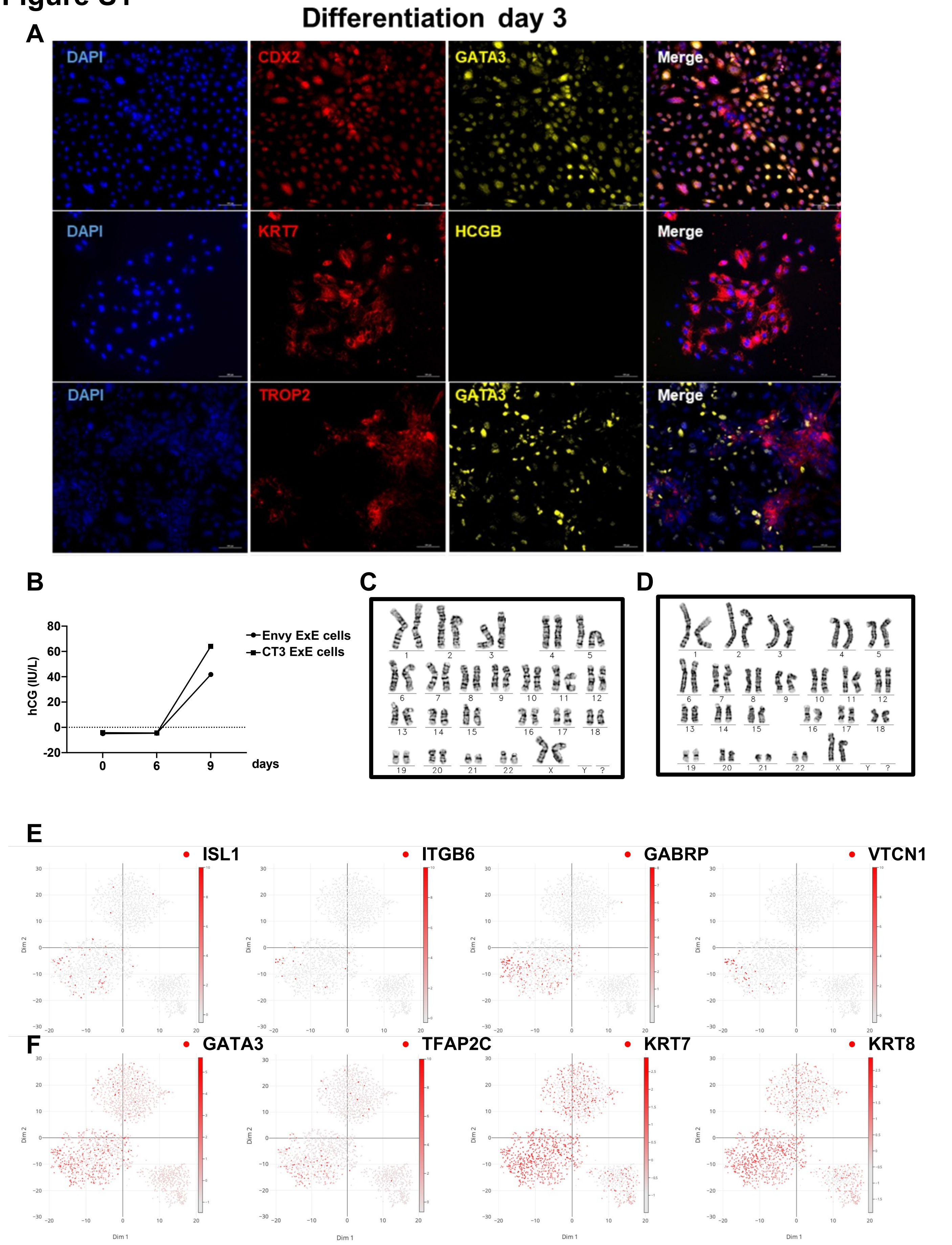
Differentiation day 3
A
DAPI
CDX2
GATA3
Merge
DAPI
KRT7
HCGB
Merge
DAPI
TROP2
GATA3
Merge
B
hCG (IU/L)
80
60
40
20
0
-20
0
6
9
days
Envy ExE cells
CT3 ExE cells
C
D
E
ISL1
ITGB6
GABRP
VTCN1
F
GATA3
TFAP2C
KRT7
KRT8
Dim 1
Dim 2

Figure S1. Characterization of ExE cells in vitro.

(A) Immunostaining for TB markers CDX2, GATA3, TROP2, KRT7, and CGB in ExE cells on differentiation day 3. DAPI counterstained the cellular nuclei. Scale bar, 50 μm.

(B) Detection of hCG in the culture of Envy hESCs during their differentiation to ExE cells at various time points.

(C) Karyotyping of Envy-hESCs.

(D) Karyotyping of CT3-hESCs.

(E) Scatter plot for amnion markers of hESCs, ExE cells and T-MSCs.

(F) Scatter plot for TB markers of hESCs, ExE cells and T-MSCs.

Figure S2

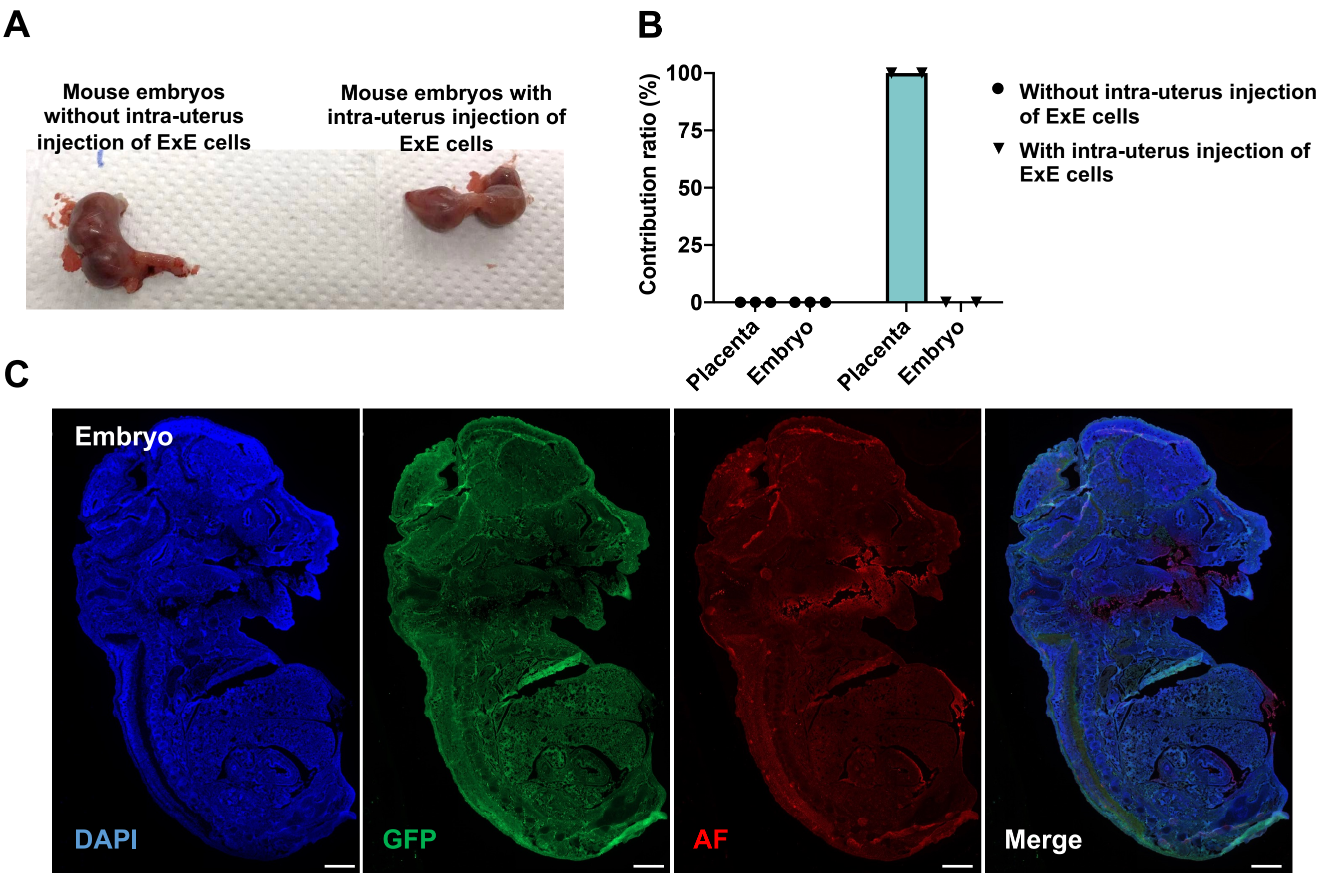
A
Mouse embryos without intra-uterus injection of ExE cells
Mouse embryos with intra-uterus injection of ExE cells
B
Contribution ratio (%)
100
75
50
25
0
Placenta
Embryo
Placenta
Embryo
Without intra-uterus injection of ExE cells
With intra-uterus injection of ExE cells
C
Embryo
DAPI
GFP
AF
Merge

Figure S2. Contribution of ExE cells to the mouse placenta but not embryo following intra-uterus delivery.

(A) Photographs of a uterine horn which contained multiple fetuses in a surrogate mouse with or without intra-uterus delivery of ExE cells.

(B) A bar chat for the ratio of ExE cell contribution to the mouse placenta or embryos following intra-uterus delivery of the cells.

(C) Immunostaining for GFP to track ExE cells in a chimeric embryo at E14. Auto fluorescence (AF) was detected as a negative control. Scale bar, 500 μm.

Figure S3

A

Cell lines	Stage of injection	Injected cells	Injected embryos	Recovered embryos	Contribution to TE	Contribution to ICM	Chimera ratio (%)
Envy	Blastocyst	ExE cells	268	221	163	0	73.76%
CT3	Blastocyst	ExE cells	56	51	37	0	72.55%

B

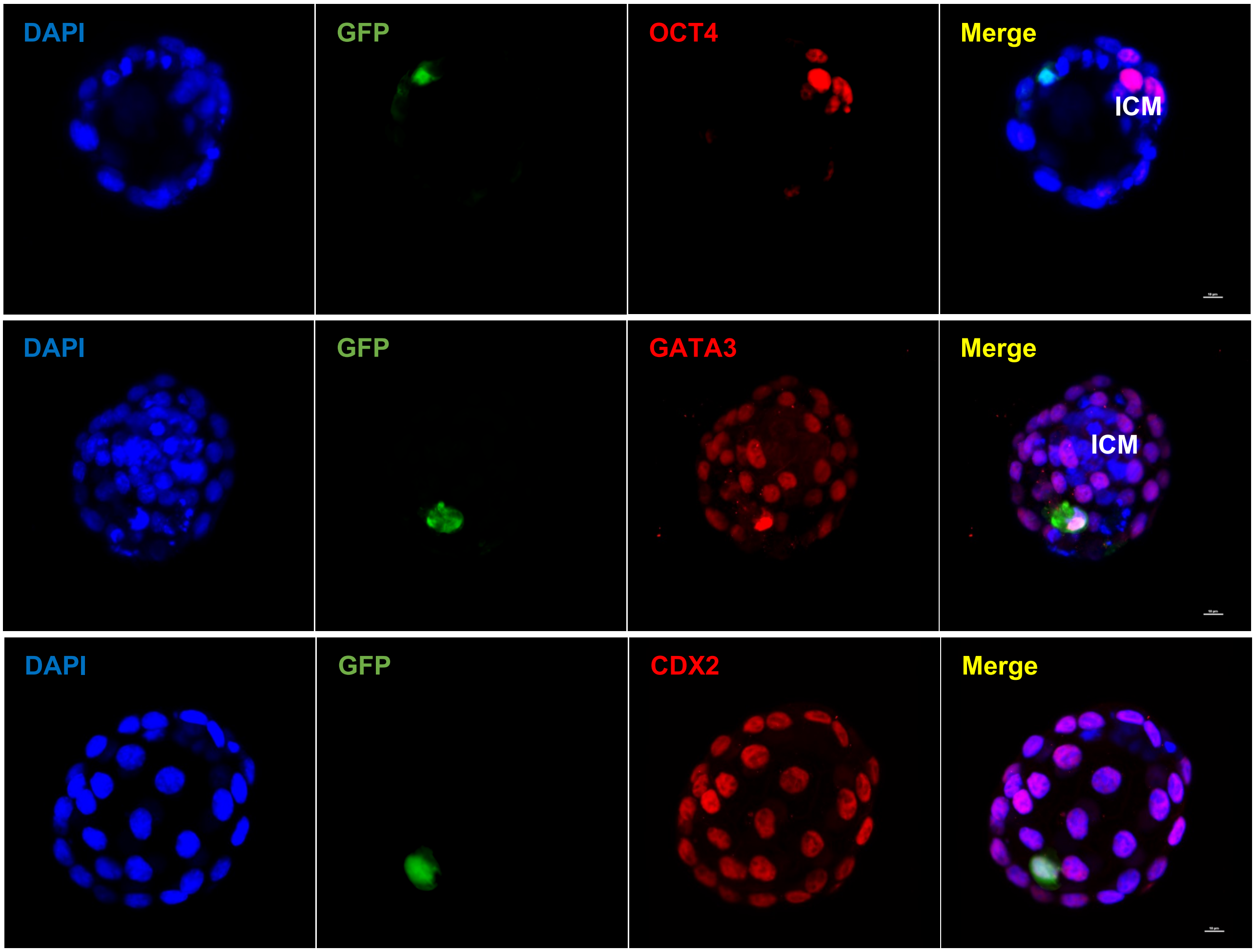

Figure S3. Contribution of ExE cells to mouse blastocysts in vitro culture.
(A) A summary for recovery of ExE cell-injected mouse blastocysts and contribution of ExE cells into the TE and ICM after 14 h in vitro culture of the injected embryos. ExE cells were differentiated from two hESC lines (Envy and CT3).
(B) Immunostaining of injected embryos for the ICM marker Oct4, TE markers Cdx2, and Gata3, and GFP for ExE cells. Scale bar, 50 µm.

Figure S4

A

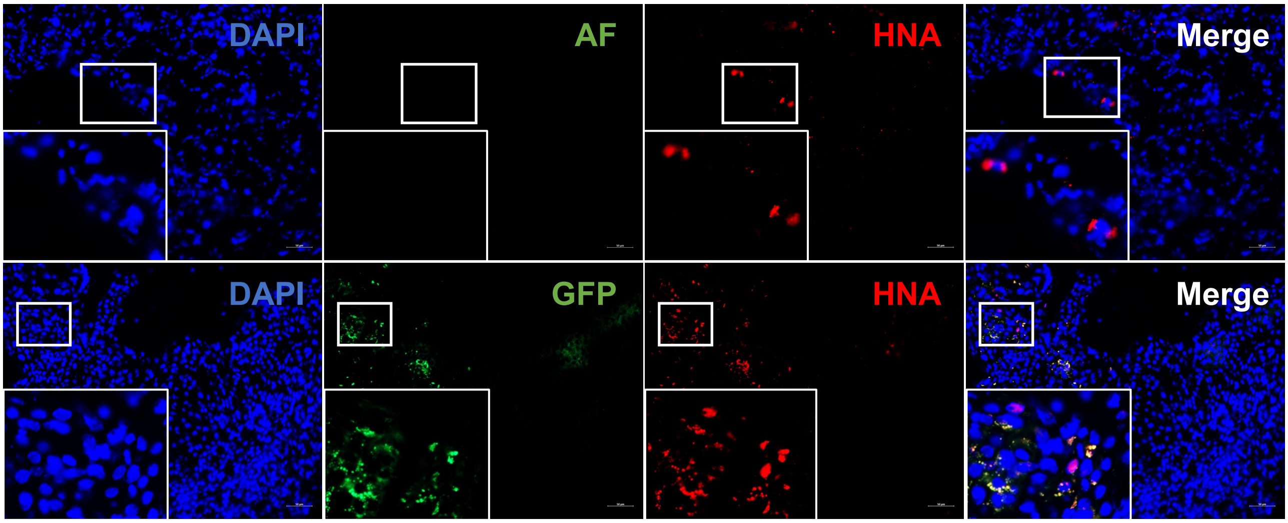
DAPI
AF
HNA
Merge
DAPI
GFP
HNA
Merge

B

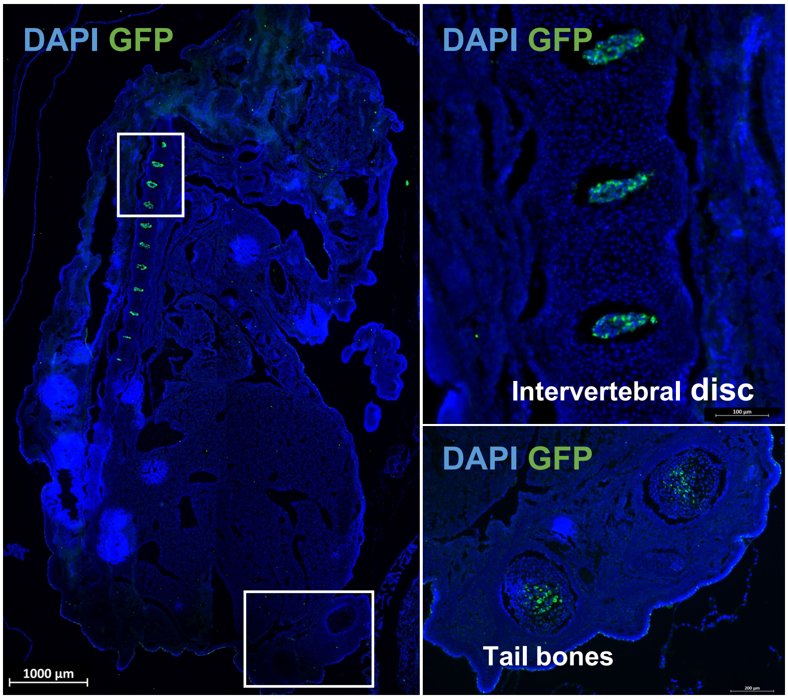
DAPI GFP
1000 µm
DAPI GFP
Intervertebral disc
DAPI GFP
Tail bones

Figure S4. Contribution of ExE cells to the mouse placenta and embryo following blastocyst injection.

(A) Immunostaining for autofluorescence (AF), GFP and HNA. DAPI counterstained the cellular nuclei. Scale bar, 50 μm.

(B) Immunostaining for GFP. DAPI counterstained the cellular nuclei. Scale bar, 50 μm.

Figure S5

A

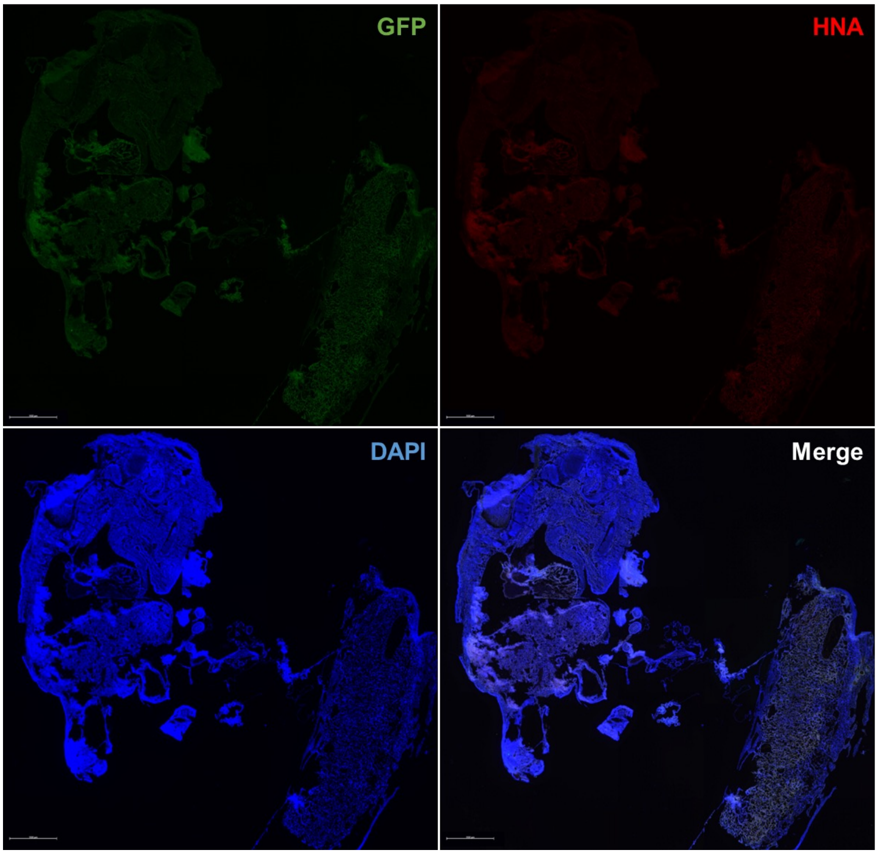
GFP
HNA
DAPI
Merge

B

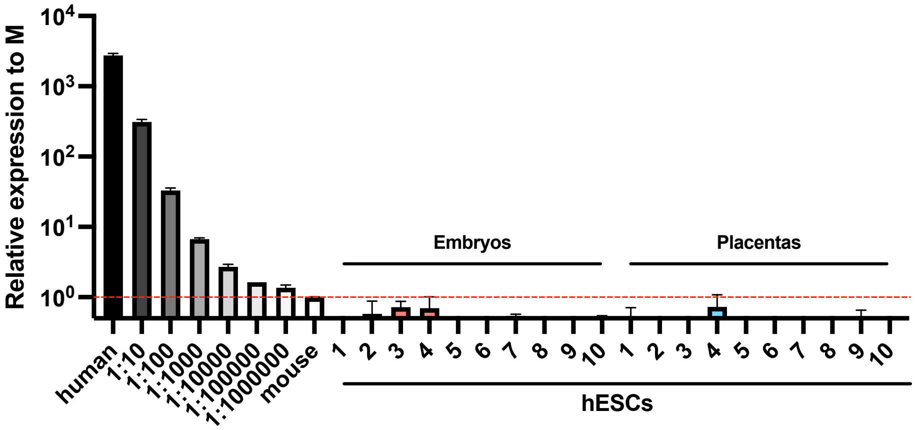
Relative expression to M
10^4
10^3
10^2
10^1
10^0
Embryos
Placentas
human
1:10
1:100
1:1000
1:10000
1:100000
1:1000000
mouse
1 2 3 4 5 6 7 8 9 10 1 2 3 4 5 6 7 8 9 10
hESCs

Figure S5. Human gDNA detection in E14 chimera formed by mouse blastocysts injected with Envy hESCs.

(A) Immunostaining for GFP and HNA. DAPI counterstained the cellular nuclei. Scale bar, 1,000 μm.

(B) QPCR detection of human gDNA in human-mouse chimeras formed by hESC-injected mouse blastocysts.
